# Psilocybin sex-dependently reduces alcohol consumption in C57BL/6J mice

**DOI:** 10.3389/fphar.2022.1074633

**Published:** 2023-01-04

**Authors:** Kenneth Alper, Janelle Cange, Ria Sah, Deanna Schreiber-Gregory, Henry Sershen, K. Yaragudri Vinod

**Affiliations:** ^1^ Department of Psychiatry, NYU Grossman School of Medicine, New York, NY, United States; ^2^ Department of Neurology, NYU Grossman School of Medicine, New York, NY, United States; ^3^ Division of Laboratory Animal Resources of Nathan Kline Institute for Psychiatric Research, Orangeburg, NY, United States; ^4^ Department of Analytical Psychopharmacology, Nathan Kline Institute for Psychiatric Research, Orangeburg, NY, United States; ^5^ Juxdapoze Statistical Consulting, Rockville, MD, United States; ^6^ Department of Neurochemistry, Nathan Kline Institute for Psychiatric Research, Orangeburg, NY, United States; ^7^ Emotional Brain Institute, Orangeburg, NY, United States; ^8^ Department of Child and Adolescent Psychiatry, NYU Grossman School of Medicine, New York, NY, United States

**Keywords:** alcohol, hallucinogen, lysergic acid diethylamide, psilocybin, psychedelic, mouse, serotonin receptor 5-HT2A agonist, substance-related and addictive disorders

## Abstract

The classical psychedelic psilocybin is of interest as a treatment for alcohol use disorder (AUD). This study investigated the effects of psilocybin on voluntary ethanol consumption in adult male and female C57BL/6J mice administered saline or psilocybin intraperitoneally as a single dose of 0.1, 0.5, 1.0 or 2.0 mg/kg and provided 20% ethanol utilizing a two-bottle choice alcohol drinking paradigm. Ethanol was provided continuously for 3 days immediately following the administration of psilocybin, then withheld for 2 days, and then provided continuously for two subsequent additional days. A multilevel model (MLM) for repeated measures was used to compare ethanol consumption and preference in psilocybin-treated groups versus controls. Ethanol consumption and preference were reduced in male mice during the 3-day interval that immediately followed psilocybin administration. The effect of psilocybin on ethanol consumption was dose-related and was consistent across the 3-day interval at dosages of 0.5 mg/kg or greater. Psilocybin had no effect on consumption or preference when ethanol was subsequently reintroduced after 2 days of withdrawal. In contrast to males, psilocybin had no significant effect on ethanol consumption or preference in female mice at any dosage or time point. The lack of an effect of psilocybin on quinine preference, and its limited interaction with locomotor activity indicated that the observed reduction in voluntary ethanol consumption was not attributable to altered taste perception or motor effects. Total fluid consumption was increased in males at some time points and psilocybin dosages and unchanged in females, and the absence of any decrease in either group at any time point indicated that the observed reduction in ethanol consumption was not mediated by nonspecific effects on consummatory behavior. The finding of a sex-dependent effect of psilocybin on ethanol consumption suggests that the C57BL/6J mouse may provide a useful experimental approach to modeling sex differences in vulnerability to AUD in addition to investigation of the neurobiological basis of the effect of classical psychedelics on alcohol drinking behavior.

## Introduction

Classical psychedelics are a focus of interest as treatment for alcohol use disorder (AUD). Lysergic acid diethylamide (LSD) (Krebs and Johansen, 2012), and subsequently psilocybin (Bogenschutz et al., 2015; Bogenschutz et al., 2022) are the classical psychedelics that have been studied most often on controlled studies in clinical settings. Collectively, observational and controlled studies indicate a prolonged effect of diminished alcohol use following the administration of single dosages of a classical psychedelic. As of this writing, a search of all studies with the term psilocybin in ClinicalTrials.gov yields a total of 120 studies, with 18 studies intending to treat substance use disorders (SUDs), eight of which focus on AUD.

Animal models may offer useful approaches to target identification and drug discovery in neurobiological investigations of psychedelics as treatment for AUD. Table 1 summarizes previously published studies reporting on the effect of the classical psychedelics psilocybin (Meinhardt et al., 2020; Meinhardt et al., 2021), LSD (Alper et al., 2018; Elsilä et al., 2022), ayahuasca (Serra et al., 2022) or 2,5-dimethoxy-4-iodoamphetamine (DOI) (Maurel et al., 1999a; Maurel et al., 1999b; Maurel et al., 2000; Oppong-Damoah et al., 2019; Berquist and Fantegrossi, 2021) on ethanol self-administration in animal models. The table also includes two serotonin 2A receptor (5-HT_2A_R) agonist psychedelic analogs produced by rational pharmaceutical design; Tabernanthalog (TBG) (Cameron et al., 2021), and (4-Bromo-3,6-dimethoxybenzocyclobuten-1-yl) methylamine hydrobromide (TCB-2) (Kimmey et al., 2022). The administered classical psychedelic or 5-HT_2A_R agonist psychedelic analog reduced ethanol consumption and/or preference in mice or rats in at least one experimental condition in all of these twelve studies, and the effect appeared dose-related in six of them (Maurel et al., 1999a; Maurel et al., 1999b; Alper et al., 2018; Berquist and Fantegrossi, 2021; Elsilä et al., 2022; Kimmey et al., 2022).

**TABLE 1:** Studies reporting on the effect of classical psychedelics and 5-HT_2A_R agonist psychedelic analogs on alcohol drinking behavior in rodents.

Study	Test drugs, dosage	Species/strain, sex	Alcohol consumption paradigm	Effect of test drug on alcohol drinking behavior
Meinhardt et al. (2021)	Psilocybin 1 or 2.5 mg/kg i.p., single dose administered 4 h prior to relapse session	Wistar rats, male	Operant ethanol self-administration, active versus inactive lever pressing in 1 h relapse session following ethanol self-administration and extinction training	Reduced ethanol self-administration 4 h following both the 1 and the 2.5 mg/kg psilocybin dosages, effect not dose-related
Meinhardt et al. (2020)	Psilocybin 0.1, 1.0, 2.5 or 10 mg/kg i.p., administered 2 to 8 times across 3 different experiments; LSD 80 and 320 μg/kg i.p., administered as an additional experimental condition in one of these experiments	Wistar rats, male and female pooled; sex evaluated as a covariate	Alcohol deprivation effect (ADE) model; dependent variable is resumption of ethanol intake following ethanol deprivation in animals previously exposed to 8 weeks of continuous ethanol availability	Reduced ethanol consumption over a 24-h interval following the second of a series of five 1.0 mg/kg psilocybin dosages administered every 12 h, with no subsequent persistent effect on ethanol consumption. No effect of psilocybin in any other experiment, including sustained effects. No effect of LSD at any dosage on ethanol consumption. No effect of psilocybin or LSD on total fluid consumption
Elsilä et al. (2022)	LSD 50 and 100 μ/kg i.p., single dose	C57BL/6J mice, male	Two-bottle choice, Intermittent access, 2 h test sessions	Reduced ethanol consumption without prolonged effects with 100 μg/kg dosage, no effect of 50 μg/kg. LSD increased water consumption, no effect on sucrose consumption
Alper et al. (2018)	LSD 25 or 50 μg/kg i.p., single dose	C57BL/6J mice, male	Two bottle choice, continuous access	Reduced ethanol consumption and preference over an interval of 46 days following administration of LSD 50 μg/kg, no significant effect of LSD 25 μg/kg. LSD had no effect on total fluid consumption
Serra et al. (2022)	Ayahuasca, lyophilized containing 0.4% N,N dimethyltryptamine (DMT) 100 mg/kg, intragastric (gavage, i.g.), administered every 3 days for one 15-day treatment period (total of 5 doses), and two additional 9 days treatment periods (total of 3 doses each) prior to respective test sessions. 5-HT_2A_R antagonist M100907 1 mg/kg i.p. preceding ayahuasca in a subgroup	Swiss mice, male	Two-bottle choice, intermittent access, test sessions began 48 h after last ayahuasca dose, presented as 15 h of ethanol access mostly during the dark phase every 2 days for a total of three 15-h sessions of ethanol access spanning a 6-day interval	Reduced ethanol consumption and preference with ayahuasca. Pretreatment with M100907 blocked the effect of ayahuasca on ethanol drinking behavior
Berquist and Fantegrossi, (2021)	DOI 0.1, 0.32, or 1 mg/kg i.p., subcutaneous, single dose	Long-Evans rats, male	Two-bottle choice, Intermittent access, 24 h test sessions	Reduced ethanol consumption and preference for at least 24 h following administration of DOI, dose-related. Total fluid consumption reduced with 0.32 mg/g DOI at postadministration time interval 0–24h, increased with 0.1 mg/kg and 0.32 mg/kg DOI at 1–4 h, no other effects on total fluid consumption with other dosages or time intervals
Oppong-Damoah et al. (2019)	DOI 3 mg/kg, i.p., single dose. 5-HT_2A_R antagonist M100907 0.1 mg/kg i.p. preceding DOI in a subgroup	Swiss-Webster mice, male stratified to high and low drinking groups	Two-bottle choice, intermittent access, 24 h test sessions	Reduced ethanol consumption at 24 h, reduced preference at 1h and 24 h following administration of DOI in the high drinking group, effect blocked by M100907. No significant effect on ethanol drinking behavior in the low drinking group. No effect of DOI on total fluid consumption in both groups
Maurel et al. (1999a)	DOI 0.3 1.0, or 3.0 mg/kg i.p., single dose. 5-HT_2A_R antagonists ritanserin 3 mg/kg or MDL100,907 0.3 mg/kg i.p. preceding DOI	cAA rats, male and female, pooled in approximately equal numbers of males and females	Two bottle choice, intermittent access 12 h test sessions during dark phase of day/night cycle	Reduced ethanol consumption and preference over 12 h post-administration interval versus 12 h baseline interval with 0.3 and 3.0 mg/kg DOI, no significant effect of 1.0 mg/kg. No effect of DOI on total fluid consumption. Pretreatment with ritanserin and MDL100,907 blocked the effect of DOI on ethanol drinking behavior
Maurel et al. (1999b)	DOI 0.1 and 0.3 mg/kg i.p., single dose	Wistar rats, male	Operant ethanol self-administration, lever pressing water vs. ethanol choice, 30-min test sessions during 12 h dark phase of day/night cycle	Reduced responding for ethanol with 0.3 mg/kg DOI, no effect of 0.1 mg/kg DOI. No effect of DOI on total fluid consumption
Maurel et al. (2000)	DOI 3 mg/kg i.p., single dose	cAA rats, male and female, pooled no mention of proportion of males versus females	Two bottle choice (ethanol, water) compared to a three bottle (ethanol, water, sucrose solution; or ethanol, water, saccharine solution) Intermittent access 24h test session	Reduced ethanol consumption with DOI. DOI increased water saccharin consumption, no effect on sucrose consumption. Concluded reduced ethanol consumption was not due to nonspecific effects on consummatory behavior
Cameron et al. (2021)	TBG 50 mg/kg i.p., single dose	C57BL/6J mice, male	Two-bottle choice, intermittent access, 48 h test sessions	Reduced ethanol consumption and preference in the first 4 h and ethanol consumption in first and second 24 h intervals following administration of TBG. No effect of TBG on total fluid consumption or sucrose preference
Kimmey et al. (2022)	TCB-2 0.1, 1.0 mg/kg i.p., single dose administered 22 h prior to each of 3 consecutive test sessions separated by intervals of 1 day	C57BL/6J mice, male, stratified by high (>50%) versus low (<50%) ethanol preference during the 4 weeks of ethanol exposure that preceded test sessions	Two bottle choice, intermittent access, 24 h test sessions	Reduced ethanol consumption and preference averaged across 3 test sessions with 1.0 mg/kg TCB-2 in mice with a high (>50%) preference for ethanol, no effect on drinking in mice with low (<50%) ethanol preference. No effect of 0.1 mg/kg TCB-2 on ethanol drinking behavior. No effect of TCB-2 on saccharin consumption. Reduced total fluid consumption due only to decreased ethanol consumption with no change in water consumption

Clinical studies of classical psychedelics for the treatment of AUD have found that the effect of diminished alcohol consumption persists beyond the time to elimination of the administered test drug. The plasma half-life of LSD in humans is 2.6 h (Dolder et al., 2017), and the elimination half-life of psilocin, the active metabolite of psilocybin is 3 h (Brown et al., 2017). A meta-analysis of controlled clinical studies of LSD for AUD found significantly greater rates of self-reported abstinence from alcohol use at 2–3 months following the administration of single dosages of LSD (Krebs and Johansen, 2012). A recent controlled study of psilocybin for AUD that compared psilocybin and psychotherapy versus placebo and psychotherapy found increased rates of alcohol abstinence and decreased days of heavy drinking at 33–36 weeks following the first of two psilocybin dosages administered 4 weeks apart (Bogenschutz et al., 2022).

Preclinical studies also indicate an effect of reduced voluntary alcohol consumption that persists beyond clearance of the classical psychedelic test drug according to the general assumption that the time interval required for metabolic clearance requires is equivalent to five elimination half-lives. A previous study found reduced ethanol consumption and preference in mice over a 46-day interval following the administration of LSD (Alper et al., 2018), which has a reported half-life in the mouse in the range of 7–37 min (Winter et al., 2005). Another study found reduced ethanol consumption and preference in mice at 24 h following the administration of DOI (Berquist and Fantegrossi, 2021), which has a reported whole blood half-life in the mouse of 1.9 h (de la Fuente Revenga et al., 2019).

Sex differences are evident regarding the clinical features of AUD as well as preclinical models of alcohol drinking behavior. Clinically, the progression of impaired functioning, negative health consequences, and entry into treatment in AUD has been reported to be more rapid among women than men, a phenomenon which has been termed “telescoping” (Hernandez-Avila et al., 2004). In mice female sex has been associated with greater ethanol consumption and preference and responding for ethanol (Rhodes et al., 2007; Becker and Koob, 2016; Sneddon et al., 2020). The only study in Table 1 that reported on sex differences found that vehicle-treated control female rats consumed more ethanol than males, although sex as a covariate was not statistically significant in the analysis for an effect of LSD and psilocybin on ethanol consumption in pooled samples of male and female animals (Meinhardt et al., 2020).

This present study follows work with LSD (Alper et al., 2018) that utilized a two-bottle choice alcohol drinking paradigm in male C57BL/6J mice, an inbred alcohol-preferring strain (Melo et al., 1996; Rhodes et al., 2007; Yoneyama et al., 2008). The present study evaluated the effect of psilocybin on alcohol drinking behavior utilizing a two-bottle paradigm in C57BL/6J mice, including its dose-dependence, persistence, and sex differences.

## Methods

**Animals:** All the experiments were conducted with adult male and female C57BL/6J mice (10–18 weeks old; Jackson Laboratory United States). Mice were given unlimited access to standard mouse chow (5001; LabDiet, St Louis, MO, United States) and water throughout the entire study. All procedures were conducted in accordance with the National Institutes of Health and Nathan Kline Institute Animal Care and Use Committee’s guidelines.

**Ethanol drinking behavior and experimental drug:** The effect of psilocybin (supplied by the Usona Institute, Madison, WI, United States) on alcohol drinking behavior was assessed using a two-bottle choice drinking paradigm. Mice were housed individually and habituated to a reverse light-dark cycle for at least 1 week. Subsequently throughout the study mice were offered a bottle containing 20% ethanol and a bottle containing water for 24 h in the beginning of the dark phase for 5 days a week on Monday through Friday, and ethanol was withheld with both bottles filled with water for 2 days on Saturday and Sunday. The positions of water and ethanol bottles were alternated every day to avoid the acquisition of place preference. Two bottles containing water and 20% ethanol were placed in a cage without a mouse to control for spillage and evaporation.

Mice were exposed to ethanol for 4 weeks to acquire ethanol consumption. On Wednesday of the following week, mice were divided into five respective groups of equal ethanol intake based on the amount of ethanol consumed on the preceding day and administered vehicle (saline) or psilocybin as a single dose of 0.1, 0.5, 1.0, or 2.0 mg/kg body weight i. p. Twenty minutes after the administration of psilocybin two bottles containing water and ethanol respectively were offered to the mice and consumption of water and ethanol were measured daily at 24-h time intervals for 3 days on Wednesday through Friday, followed by a 2-day withdrawal period, with subsequent resumption of ethanol availability for two more days on Monday and Tuesday to maintain a schedule of ethanol exposure similar to that used during the acquisition of ethanol consumption.

**Quinine preference test:** Quinine preference was assessed using a two-bottle choice drinking paradigm. Mice were habituated to two bottles, one containing water and one containing quinine (0.06 mM) for a week. Mice were then administered either vehicle (saline) or psilocybin (0.5, 1.0, or 2.0 mg/kg body weight, i. p.) once before the onset of the dark cycle. Bottles containing quinine and water were offered 20 min posttreatment and consumption of water and quinine were measured for daily for 3 days, and preference for quinine was calculated. The positions of the water and quinine bottles were alternated every day to avoid place preference. Data were corrected for the loss of liquid due to spillage and evaporation by comparing weights of water and quinine bottles in a control cage without a mouse.

**Locomotor activity:** Ambulatory activity (interruption of the total number of beams on both the *x* and *y*-axis), and distance travelled were measured with an infrared beam-based activity sensor (ATM3; Columbus Instrument, Columbus, OH, United States) over a 24-h period beginning at the dark cycle following administration of either vehicle (saline) or psilocybin (0.5, 1.0, and 2.0 mg/kg body weight, i. p.) to alcohol-naïve mice.

**Statistical analysis:** Statistical analyses were completed in SAS software Version 9.4. The Satterthwaite t-test was utilized for univariate comparisons. A multi-level mixed effects model (MLM) approach was chosen for the repeated measures model (Beckman et al., 1987) and analyses performed using the MIXED procedure (SAS Institute Inc., 2022) with a *p*-value ≤ 0.05 as a threshold for significance. All MLMs included the independent variables of time, psilocybin treatment, and time by treatment interaction.

MLM was selected as a repeated measures model that controls for nested random effects at the level of individual mice within groups defined by the fixed effect of psilocybin dosage, as well as autoregressive random effects which are cyclical over time and could confound the detection of a fixed treatment effect.

No violations of MLM assumptions were detected, including non-normally distributed residuals, heterogeneity of residual variance, and nonlinearity between the independent and dependent variables. The Durbin-Watson test for autocorrelation indicated significant first- and second-order autocorrelation for consumption and preference for both males and females (all *p* < 0.0001), which led us to control for autoregression in the MLM. Model fit statistics were evaluated throughout the analysis and included Akaike’s Information Criterion (AIC) and Bayesian Information Criterion (BIC), and the Cox-Snell adjusted R-square. Additions to the model, specifically the time x treatment interaction and controlling for autoregressive structure reduced BIC and AIC, indicating that these additions improved the goodness of fit, as well as the extent to which the model explained the variance in the dependent variable as indicated by the Cox-Snell adjusted R-square.

## Results

**FIGURE 1:**
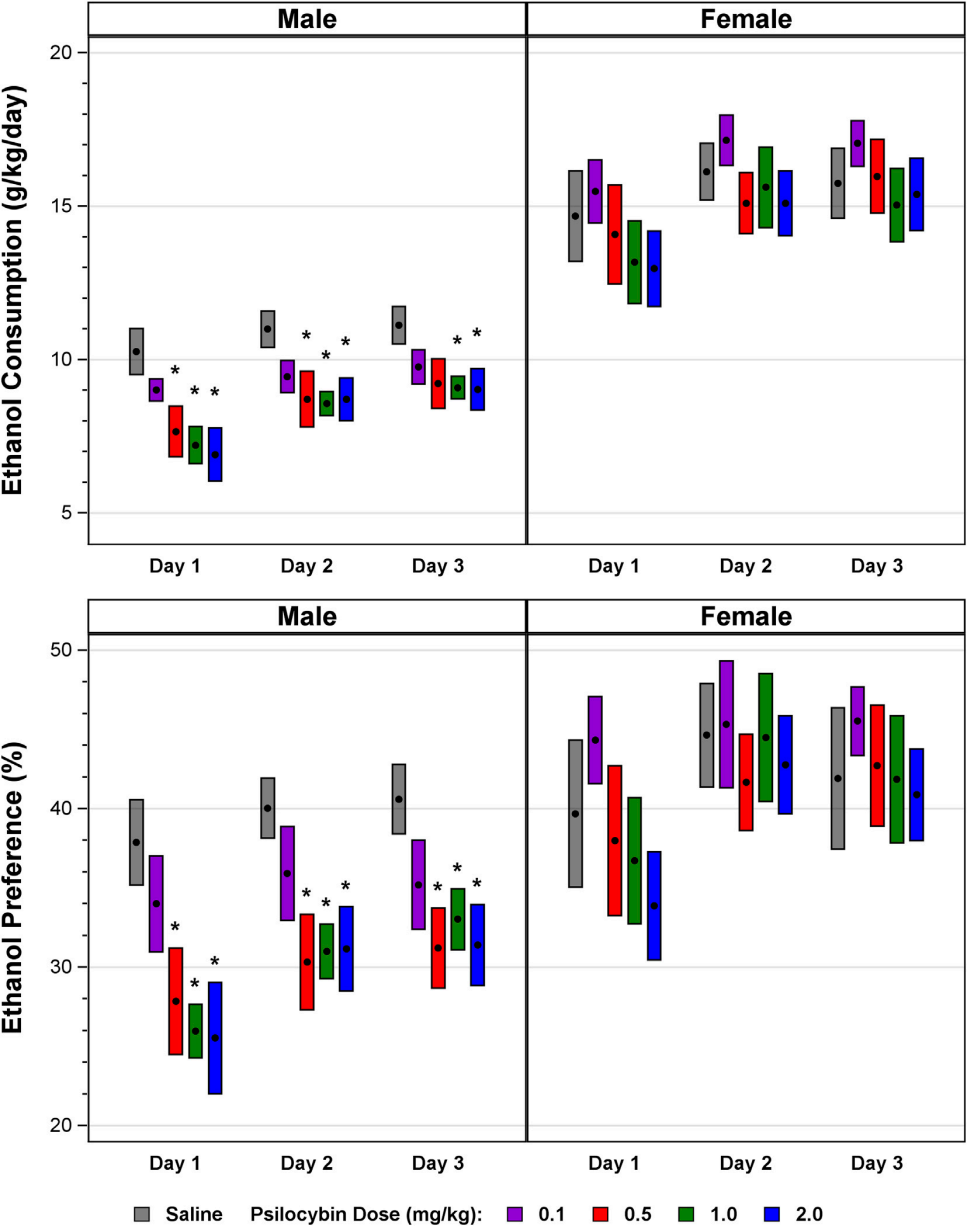
Mean daily ethanol consumption and preference in male and female mice across the 3-day interval following a single psilocybin dosage of 0.1, 0.5, 1.0, or 2.0 mg/kg. Twenty minutes after the administration of psilocybin or saline mice were provided continuous access to a bottle containing 20% ethanol and a bottle containing water, and consumption of water and ethanol were measured daily at 24-h time intervals for 3 days. The black solid circle at the center of each bar is the mean, centered in a bar with a vertical length from −1 to 1 standard error of the mean. * *p* ≤ 0.05, Satterthwaite t-test.

**Ethanol consumption and preference:** Figure 1 plots mean daily ethanol consumption and preference for 3 days immediately following a single psilocybin dosage of 0.1, 0.5, 1.0, or 2.0 mg/kg administered to male and female mice. The preliminary statistical analysis represented in Figure 1 is limited to univariate comparison of the means for consumption and preference in psilocybin-treated versus control mice. The univariate results in males indicate significant effects of reduced ethanol consumption or preference across the 3 days of observation for psilocybin dosages of 0.5, 1.0, and 2.0 mg/kg. The effect of psilocybin on alcohol drinking behavior in male mice appears to be dose-related, no univariate comparison to control with the 0.1 mg/kg psilocybin dosage reached significance. For female mice, in contrast to the males, none of the univariate comparison were significant.

As presented in Table 2, MLM statistics indicate a significant effect of psilocybin on alcohol consumption and preference in male mice that is sustained across the 3-day interval immediately following psilocybin administration. In contrast to the males, MLM analysis found no significant effect of psilocybin on alcohol drinking behavior in females. The effect of psilocybin on ethanol consumption and preference in male mice appears to be dose related; in Table 2 only one of the four comparisons restricted to the 0.1 mg/kg psilocybin dosage in male mice was significant, in contrast to the significant results for comparisons involving the 0.5, 1.0, or 2.0 mg/kg psilocybin dosages. The effect of psilocybin on ethanol consumption and preference in males for days 2–3 only was significant, indicating that the significant results for ethanol consumption and preference in males for days 1–3 were not accounted for by treatment effect limited only to day 1.

**TABLE 2:** MLM statistics evaluating the effect of psilocybin on ethanol consumption and preference versus vehicle-only controls in male and female C57BL/6J mice. Psilocybin was administered as a single dosage of 0.1, 0.5, 1 or 2 mg/kg. At 20 min posttreatment mice were provided continuous access to a bottle containing 20% ethanol and a bottle containing water, and consumption of water and ethanol were measured daily at 24-h time intervals for 3 days. MLM analysis included each of the four respective psilocybin dosages individually, and all four dosages collectively (“0.1, 0.5, 1, and 2 mg/kg”). MLM statistics were computed for days 1–3 collectively (“Days 1, 2, and 3”), and also for only days 2–3 collectively (“Days 2 and 3”) to address the possibility that a sufficiently large treatment effect limited only to day 1 could have yielded a significant MLM result for days 1–3.

Psilocybin Dosage	Males				Females			
	Ethanol consumption		Ethanol preference		Ethanol consumption		Ethanol preference	
	Days 1, 2, and 3	Days 2 and 3	Days 1, 2, and 3	Days 2 and 3	Days 1, 2, and 3	Days 2 and 3	Days 1, 2, and 3	Days 2 and 3
	F, df, p							
0.1 mg/kg	4.38, 1, 0.0500*	4.00, 1, 0.0599	2.17, 1, 0.1569	2.42, 1, 0.1365	0.60, 1, 0.4482	0.93, 1, 0.3462	0.38, 1, 0.5454	0.20, 1, 0.6569
0.5 mg/kg	5.52, 1, 0.0291*	4.51, 1, 0.0463*	8.31, 1, 0.0092*	8.92, 1, 0.0073*	0.08, 1, 0.7807	0.08, 1, 0.7820	0.06, 1, 0.8075	0.05, 1, 0.8278
1.0 mg/kg	15.25, 1, 0.0009*	13.69, 1, 0.0014*	15.77, 1, 0.0008*	12.76, 1, 0.0019*	0.30, 1, 0.5903	0.15, 1,0.6996	0.04, 1, 0.8464	0.00, 1, 0.9849
2.0 mg/kg	8.61, 1, 0.0085*	6.96, 1, 0.0162*	9.12, 1, 0.0070*	9.00, 1, 0.0074*	0.43, 1, 0.5185	0.23, 1, 0.6382	0.38, 1, 0.5425	0.10, 1, 0.7582
0.1, 0.5, 1.0, and 2.0 mg/kg	3.73, 4, 0.0101*	2.78, 4, 0.0372*	3.89, 4, 0.0082*	3.34, 4, 0.0173*	0.57, 4, 0.6877	0.53, 4, 0.7114	0.40, 4, 0.8068	0.17, 4, 0.9530

*p ≤ 0.05.

After the 3 days immediately following the administration of psilocybin (Figure 1; Table 2), ethanol was withheld for 2 days, and then subsequently provided with continuous access for two additional days. In males during this 2-day interval of resumed ethanol availability, psilocybin no longer had a significant effect on ethanol consumption (F = 0.18, df = 4, *p* = 0.9452 for all psilocybin dosages collectively) or preference (F = 0.37, df = 4, *p* = 0.8270 for all psilocybin dosages collectively). In females during this 2-day interval of resumed ethanol availability, psilocybin remained without a significant effect on ethanol consumption (F = 0.09, df = 4, *p* = 0.9863 for all psilocybin dosages collectively) or preference (F = 0.52, df = 4, *p* = 0.7214 for all dosages collectively).

Control vehicle-treated female mice consumed more ethanol than control males. For the control groups averaged across the 3 days immediately following the administration of psilocybin, mean ethanol consumption for females was 15.52 ± 3.90 g/kg/day versus 10.80 ± 2.15 g/kg/day for males (t = 6.10, df = 49.64, *p* < 0.0001). Mean ethanol preference in control females was 42.08 ± 13.55% versus 39.50 ± 7.40% for control males (t = 0.96, df = 49.64, *p* = 0.3435).

**FIGURE 2:**
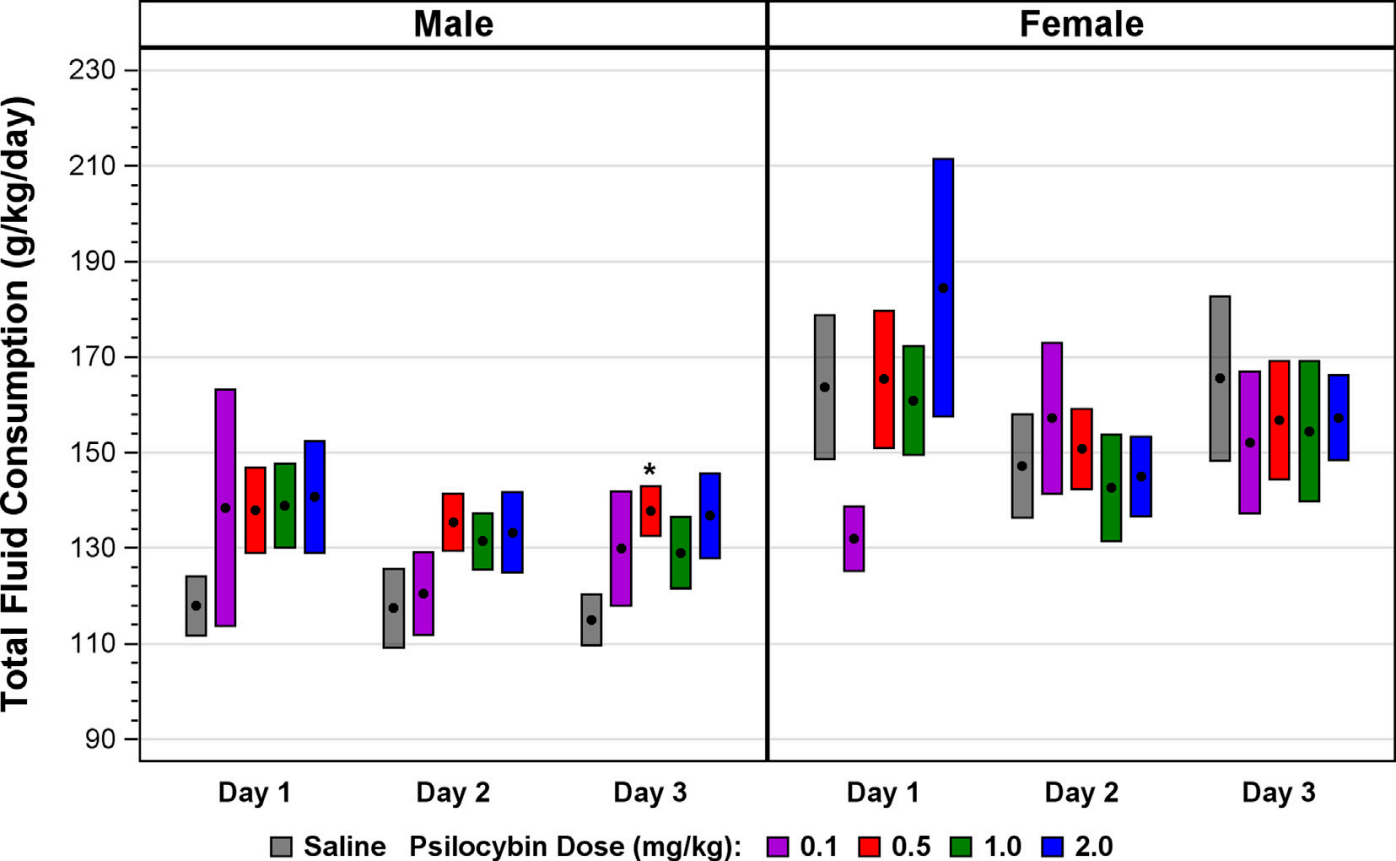
Total fluid consumption across the 3-day interval following a single psilocybin dosage of 0.1, 0.5, 1.0, or 2.0 mg/kg. The black solid circle at the center of each bar is the mean, centered in a bar with a vertical length from −1 to 1 standard error of the mean.*p ≤ 0.05, Satterthwaite t-test.

**Total fluid consumption:** The effect of psilocybin on total fluid consumption was limited to increases at some time points and dosages in males, with no effect in females (Figure 2; Table 3). The effect of psilocybin on total fluid consumption in males for all psilocybin dosages collectively on days 1–3 is not significant, in contrast to the highly significant effect of psilocybin on ethanol consumption and preference for all dosages collectively in males for days 1–3 (Table 2). Only the effects of 0.5 mg/kg and 1.0 mg/kg dosages across days 1–3 in males reached significance, and the effect of the 1.0 mg/kg dosage for days 2–3 was not significant, suggesting that the effect on day 1 drove the significance of the statistic for days 1–3. This contrasts with the significant effect of psilocybin on ethanol consumption and preference in males for days 2–3. An increase in total fluid consumption together with decreased ethanol consumption indicates greater water consumption, which could have accentuated the significance of the result for ethanol preference, but does not explain the finding of reduced ethanol consumption. The absence of any decrease in total fluid consumption at any time point or psilocybin dosage indicates that nonspecific effects on consummatory behavior do not account for the observed reduction in ethanol consumption.

**TABLE 3:** MLM statistics evaluating the effect of psilocybin on total fluid consumption versus vehicle-only controls in male and female C57BL/6J mice. MLM analysis included each of the four respective psilocybin dosages individually, and all four dosages collectively. MLM statistics were computed for days 2–3 to address the possibility that a sufficiently large treatment effect limited only to day 1 could have yielded a significant MLM result for days 1–3. *p ≤ 0.05.

Psilocybin Dosage	Total fluid consumption			
	Males		Females	
	Days 1, 2, and 3	Days 2 and 3	Days 1, 2, and 3	Days 2 and 3
	F, df, p			
0.1 mg/kg	1.01, 1, 0.3275	0.67, 1, 0.2856	0.43, 1, 0.5214	0.01, 1, 0.9321
0.5 mg/kg	6.40, 1, 0.0199*	6.52, 1, 0.0189*	0.01, 1, 0.9425	0.03, 1, 0.8734
1.0 mg/kg	5.01, 1, 0.0368*	2.63, 1, 0.1203	0.12, 1, 0.7326	0.18, 1, 0.6744
2.0 mg/kg	3.94, 1, 0.0617	3.49, 1, 0.0774	0.04, 1, 0.8372	0.11, 1, 0.7412
0.1, 0.5, 1, and 2 mg/kg	1.23, 4, 0.3126	1.46, 4, 0.2295	0.26, 4, 0.9025	0.07, 4, 0.9908

**FIGURE 3:**
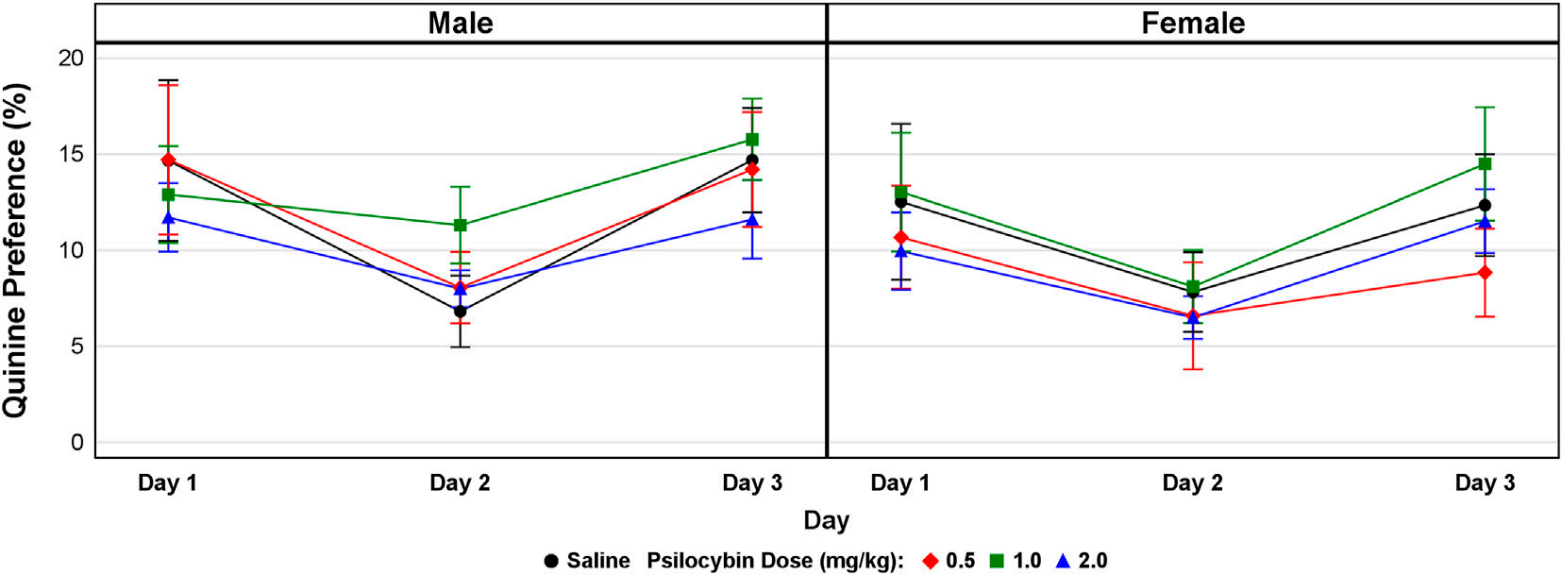
Quinine preference assessed using a two-bottle choice drinking paradigm with bottles containing water and quinine (0.06 mM). Mice were administered either vehicle (saline) or psilocybin (0.5, 1.0, or 2.0 mg/kg i.p.) and consumption of water and quinine were measured daily for 3 days.

**FIGURE 4:**
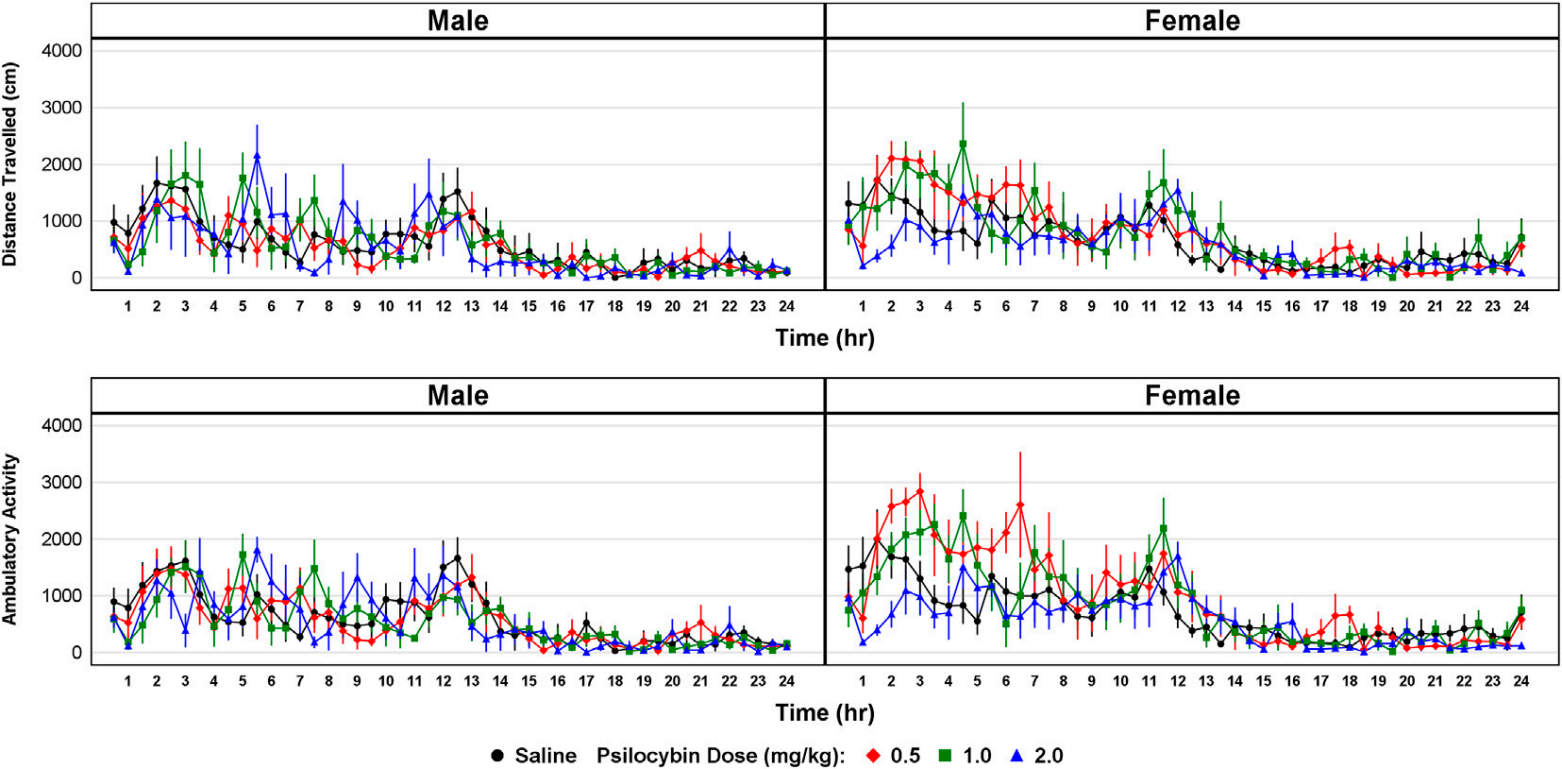
Distance travelled (upper panel) measured with an infrared beam-based activity sensor and ambulatory activity (lower panel) measured as interruptions of the total number of beams on both the *x* and *y*-axis over a 24-h period beginning at the dark cycle following administration of either vehicle (saline) or psilocybin (0.5, 1.0 or 2.0 mg/kg i.p.) to alcohol-naïve mice.

**Quinine preference and locomotor activity:** As presented in Figure 3, psilocybin had no effect on quinine preference in both males (F = 0.43, df = 3, *p* = 0.734) and females (F = 0.50, df = 3, *p* = 0.685) indicating that the effect of psilocybin on voluntary ethanol consumption does not appear to have been mediated by altered peripheral taste perception. The effect of psilocybin on ambulatory counts in male mice was not significant (F = 0.158, df = 3, *p* = 0.923), but was significant in females (F = 4.316, df = 3, *p* = 0.013). As indicated in Figure 4, the significant effect on ambulatory counts appears to have been driven by larger counts for the 0.5 mg/kg dosage during the first 8 h of the 24-h period of observation. The effect of psilocybin on distance travelled was not significant in males (F = 0.128, df = 3, *p* = 0.943) or females (F = 1.412, df = 3, *p* = 0.261). Overall, the locomotor activity data indicate that neither the finding of a significant effect of psilocybin on ethanol consumption in males nor the lack of a significant effect in females appear to be explainable on the basis of motor effects.

## Discussion

This study found that psilocybin administered as a single dose reduced ethanol consumption and preference in a two-bottle choice drinking paradigm in male C57BL/6J mice. The effect was dose-related and persisted for 3 days following psilocybin administration. The effect was sex-dependent, psilocybin did not affect ethanol consumption or preference in female mice. The results reported here are in general agreement with other preclinical studies reporting that classical psychedelics reduce voluntary alcohol consumption in rodents (Table 1).

The reduced ethanol consumption and preference observed in male mice administered psilocybin appears to have persisted beyond its metabolic clearance. The reported half-life of psilocin, the active metabolite of psilocybin is approximately 30 min in mice (Horita and Weber, 1962). This finding is consistent with other studies in Table 1 that also suggest an effect of reduced alcohol drinking behavior by a classical psychedelic that persists beyond its metabolic clearance (Alper et al., 2018; Berquist and Fantegrossi, 2021). In the present study psilocybin reduced ethanol consumption in males for 3 days, with no further effect after ethanol was subsequently withheld for two additional days. In contrast to the present work with psilocybin, in a previous study the effect of LSD on ethanol consumption persisted repeatedly over a similar 2-day withdrawal period for 6 weeks (Alper et al., 2018), which might indicate some distinction among the targets or downstream signaling characteristics of these respective classical psychedelics (Nichols, 2016). Future work should determine if the effect of psilocybin might be modified when ethanol is provided continuously or withdrawn for a longer time interval. From a translational perspective, the duration of the effect of psilocybin beyond its metabolic clearance appears consistent with clinical evidence for persistent treatment effects of psilocybin in AUD (Bogenschutz et al., 2015; Bogenschutz et al., 2022) as well as other SUDs including nicotine use disorder (Johnson et al., 2017), and mood and anxiety disorders (Ross et al., 2016; Carhart-Harris et al., 2021).

The finding of a persistent effect of psilocybin on alcohol consumption differs from a previous study that evaluated psilocybin and LSD in a rat model of relapse utilizing re-exposure following alcohol deprivation in animals with previously established alcohol consumption (Meinhardt et al., 2020) (Table 1). That study, in which psilocybin was administered according to three dosage protocols including repeated “microdosing”, found only a reduction in ethanol consumption for 24 h following the second of two 1 mg/kg dosages of psilocybin administered 12 h apart, and no other significant effects of psilocybin or LSD on ethanol consumption were observed for other time points or experiments. Differing rodent species and ethanol self-administration protocols in the above study might explain the divergent findings regarding persistent effects of psilocybin in this present study, as well as our previous work with LSD (Alper et al., 2018). Continuous access ethanol administration distinguishes our work from the other studies in Table 1, which aimed to model binge drinking utilizing a relapse model (Meinhardt et al., 2020) or intermittent access (Oppong-Damoah et al., 2019; Berquist and Fantegrossi, 2021; Elsilä et al., 2022). Continuous access is arguably a valid preclinical model of AUD, which is often expressed in humans in settings of continuous alcohol availability with consummatory behaviors constrained only by the need to manage interpersonal, occupational or financial strain and the toxic effects of alcohol itself.

The present study is apparently the first preclinical study to directly compare the effect of a classical psychedelic on alcohol self-administration in male versus female animals. The finding of a treatment effect in male but not female C57BL/6J mice appears consistent with findings reported elsewhere that relative to males, female mice consume greater quantities of ethanol and are more resistant to treatment effects (Rhodes et al., 2007; Becker and Koob, 2016; Sneddon et al., 2020). Female mice also display more motivational salience of ethanol as evidenced by more persistent consumption in the presence of aversive stimuli such as the addition of quinine (Sneddon et al., 2020). The lack of an effect of psilocybin on ethanol consumption in female mice reported here may suggest a general correspondence with the accelerated development of alcohol-related problems among women with AUD (Hernandez-Avila et al., 2004). If replicated, the sex-dependent findings reported here suggest the utility of preclinical investigative approaches involving the administration of sex hormones or gonadectomy.

Future research should utilize pharmacokinetic analysis and the administration of higher dosages of psilocybin to evaluate whether the rate of metabolism of psilocybin might explain its apparent lack of effect on ethanol consumption in females observed in this study. A recent study that found equivalent dosages of DOI administered to C57BL/6J mice produced lower concentrations in plasma and brain tissue in females compared to males (Jaster et al., 2022) suggests this possibility. Interestingly, in that study the head twitch response (HTR) was increased in female mice, and not decreased as might be expected based on accelerated metabolism of DOI, suggesting additional factors such as receptor number and occupancy (Jaster et al., 2021) or post-receptor signaling and interactions with receptors other than the 5-HT_2A_R (Nichols, 2016; Hesselgrave et al., 2021) may also be involved in sex-dependent differences in classical psychedelic effects.

In the present study ethanol consumption was measured at 24-h intervals, which does not exclude the possibility of an effect in females present earlier in the drinking session on day 1. The AUD phenotype to be modelled may provide a useful approach to studying the relationship of sex-dependent differences in treatment response to baseline level of ethanol consumption. Administration of ethanol as vapor, a model of heavy drinking, increases ethanol consumption in the C57BL/6J mouse (Becker and Lopez, 2004) and could address an experimental question of whether psilocybin remains effective in heavy drinking and dependence.

Psilocybin did not affect quinine preference, which weighs against the possible confound that altered peripheral taste sensation might have mediated the observed reduction of ethanol consumption. Among published studies of the effect of classical psychedelics or 5-HT_2A_R psychedelic analogs on alcohol drinking behavior in rodents (Table 1), the present study is apparently the first to address this possibility.

An alternative possibility, that non-specific effects on consummatory behaviors might explain reductions in alcohol drinking behaviors observed with classical psychedelics in rodent models has been more extensively investigated. Consistent with the results reported here, studies of classical psychedelics or 5-HT_2A_R psychedelic analogs utilizing rodent alcohol drinking paradigms have found modest increases in total fluid or water consumption (Elsilä et al., 2022) or no change (Maurel et al., 1999a; Maurel et al., 1999b; Maurel et al., 2000; Alper et al., 2018; Oppong-Damoah et al., 2019; Meinhardt et al., 2020; Cameron et al., 2021; Kimmey et al., 2022; Serra et al., 2022). Additional reports of no reduction in sucrose or saccharin preference (Maurel et al., 2000; Cameron et al., 2021; Elsilä et al., 2022; Kimmey et al., 2022), or change in intracranial self-stimulation current-intensity thresholds (Elsilä et al., 2022), and another study in which psilocybin restored sucrose preference in chronically stressed C57BL/6J mice (Hesselgrave et al., 2021), weigh against the possibility that nonspecific effects on appetitive behavior or reward could explain the reported reductions in alcohol consumption observed with classical psychedelics or 5-HT_2A_R psychedelic analogs. Collectively, the experimental evidence appears to indicate that the effect of classical psychedelics on reducing ethanol consumption is not due to nonspecific effects on consummatory behavior.

In addition to the need for independent replication, a limitation of this work is the need to extend to the investigation of the mechanistic basis of the apparent effect of classical psychedelics on alcohol drinking behavior. Classical psychedelics have been termed “neuroplastogens” (Olson, 2022) in view of persistent effects that apparently involve interactions with neurotrophins and transcription factors to encode epigenetic landscapes (de la Fuente Revenga et al., 2021), which may provide a domain of variables to investigate the mechanistic basis of apparent persistent effects of classical psychedelics on voluntary ethanol consumption.

Classical psychedelics are strong 5-HT_2A_R agonists, and the action of classical psychedelics appears to require other additional signaling elements such as metabotropic glutamate receptor 2 (mGlu2R) (Meinhardt et al., 2021) and formation of 5-HT_2A_R-mGlu2R dimer (Moreno et al., 2011). Classical psychedelics produce 5-HT_2A_R desensitization, which may enhance signaling *via* mGluR2 in view of the reciprocally inhibitory relationship between the 5-HT_2A_R and mGluR2 (Nichols, 2016). Antagonism of mGluR2 increases voluntary ethanol consumption in the rat (Zhou et al., 2013), and psilocybin enhances the expression the gene encoding the mGlu2R in the rat, and this effect was blocked with by the 5-HT_2A_R antagonist ketanserin in a study that also reported reduced operant ethanol self-administration in rats treated with psilocybin (Meinhardt et al., 2021). These results appear consistent with an older report that 5-HT_2A_R antagonists diminish the effect of DOI in reducing ethanol consumption in rats (Maurel et al., 1999a).

Classical psychedelics mediate characteristic effects in diverse species that include the HTR in mice (Halberstadt and Geyer, 2013), a discriminable stimulus in rats (Nichols, 2016), and subjective states attributed with spiritual, transcendent and ineffable qualities in humans (Griffiths et al., 2006). The potencies with which specific classical psychedelics produce these respective effects in mice, rats and humans are strongly correlated across species (Halberstadt et al., 2020), suggesting a possibility of some degree of communality regarding their underlying neurobiological basis. Along with the results found in this study, reports that classical psychedelics reduce alcohol consumption in mice (Alper et al., 2018; Oppong-Damoah et al., 2019; Cameron et al., 2021; Elsilä et al., 2022; Kimmey et al., 2022; Serra et al., 2022), rats (Maurel et al., 1999a; Maurel et al., 1999b; Maurel et al., 2000; Meinhardt et al., 2020; Berquist and Fantegrossi, 2021; Meinhardt et al., 2021), and humans (Krebs and Johansen, 2012; Bogenschutz et al., 2015; Bogenschutz et al., 2022) may also suggest the possible involvement of common targets. The threshold dosages at which we have found consistent effects on alcohol consumption and preference in male C57BL/6J mice are 50 μg/kg for LSD (Alper et al., 2018), and in this study, 0.5 mg/kg for psilocybin. These dosages are similar to the respective reported values for mouse HTR ED_50_ of 52.9 μg/kg for LSD and 0.38 mg/kg for psilocybin (Halberstadt et al., 2020). The mouse HTR may provide a contributory approach to the identification of common targets linking psychedelic effects to the mechanistic basis of the apparent effect of classical psychedelics on voluntary alcohol consumption.

In conclusion, we report here that a single dose of psilocybin dose-dependently reduces ethanol consumption in male but not female C57BL/6J mice over a time interval that apparently extends beyond the elimination of the drug. The apparent sex-dependence of the effect of psilocybin on voluntary alcohol consumption supports the C57BL/6J mouse model as a potential experimental approach to modeling sex differences in vulnerability to AUD. These results suggest that animal models merit further study as an approach to neurobiological investigation and the identification of targets towards drug discovery and the development of classical psychedelics as pharmacotherapy for the treatment of SUDs.

## Data Availability

The raw data supporting the conclusions of this article will be made available by the authors, without undue reservation.
